# Genome-wide association study of corticobasal degeneration identifies risk variants shared with progressive supranuclear palsy

**DOI:** 10.1038/ncomms8247

**Published:** 2015-06-16

**Authors:** Naomi Kouri, Owen A. Ross, Beth Dombroski, Curtis S. Younkin, Daniel J. Serie, Alexandra Soto-Ortolaza, Matthew Baker, Ni Cole A. Finch, Hyejin Yoon, Jungsu Kim, Shinsuke Fujioka, Catriona A. McLean, Bernardino Ghetti, Salvatore Spina, Laura B. Cantwell, Martin R. Farlow, Jordan Grafman, Edward D. Huey, Mi Ryung Han, Sherry Beecher, Evan T. Geller, Hans A. Kretzschmar, Sigrun Roeber, Marla Gearing, Jorge L. Juncos, Jean Paul G. Vonsattel, Vivianna M. Van Deerlin, Murray Grossman, Howard I. Hurtig, Rachel G. Gross, Steven E. Arnold, John Q. Trojanowski, Virginia M. Lee, Gregor K. Wenning, Charles L. White, Günter U. Höglinger, Ulrich Müller, Bernie Devlin, Lawrence I. Golbe, Julia Crook, Joseph E. Parisi, Bradley F. Boeve, Keith A. Josephs, Zbigniew K. Wszolek, Ryan J. Uitti, Neill R. Graff-Radford, Irene Litvan, Steven G. Younkin, Li-San Wang, Nilüfer Ertekin-Taner, Rosa Rademakers, Hakon Hakonarsen, Gerard D. Schellenberg, Dennis W. Dickson

**Affiliations:** 1 Department of Neuroscience, Mayo Clinic College of Medicine, Jacksonville, Florida 32224, USA; 2 Department of Pathology and Laboratory Medicine, Perelman School of Medicine, University of Pennsylvania, Philadelphia, Pennsylvania 19104, USA; 3 Division of Biomedical Statistics and Informatics, Department of Health Sciences Research, Mayo Clinic College of Medicine, Jacksonville, Florida 32224, USA; 4 Victorian Brain Bank Network, Mental Health Research Institute, Parksville, Victoria 3052, Australia; 5 Department of Pathology and Laboratory Medicine, Indiana University School of Medicine, Indianapolis, Indiana 46202, USA; 6 Department of Neurology, Indiana University School of Medicine, Indianapolis, Indiana 46202, USA; 7 Cognitive Neuroscience Laboratory, Brain Injury Research, Rehabilitation Institute of Chicago, Chicago, Illinois 60611, USA; 8 Department of Physical Medicine and Rehabilitation, Northwestern University, Illinois 60208, USA; 9 Departments of Psychiatry and Neurology, Columbia University, New York, New York10027, USA; 10 Institut for Neuropathology and Prion Research and Brain Net Germany, Ludwig-Maximilians-Universität, Munich 80539, Germany; 11 Department of Pathology and Laboratory Medicine, Emory University School of Medicine, Atlanta, Georgia 30307, USA; 12 Department of Neurology, Emory University, Atlanta, Georgia 30307, USA; 13 Department of Pathology and the Taub Institute for Research on Alzheimer’s disease and the Aging Brain, Columbia University, New York, New York 10027, USA; 14 Department of Neurology, University of Pennsylvania Health System, Philadelphia, Pennsylvania 19104, USA; 15 Department of Psychiatry, Center for Neurobiology and Behavior, University of Pennsylvania School of Medicine, Philadelphia, Pennsylvania 19104, USA; 16 Department of Neurology, Innsbruck Medical University, Innsbruck 6020, Austria; 17 Department of Pathology, University of Texas Southwestern Medical Center, Dallas, Texas 75235, USA; 18 Department of Neurology, Technical University Munich, 81377 Munich, Germany; 19 Department of Translational Neurodegeneration, German Center for Neurodegenerative Diseases (DZNE), 81677 Munich, Germany; 20 Department of Neurology, Philipps University, 35033 Marburg, Germany; 21 Institut for Humangenetik, Justus-Liebig-Universität, Giessen 35390, Germany; 22 Department of Human Genetics, University of Pittsburgh, Pittsburg, Pennsylvania 15260, USA; 23 Department of Neurology, Rutgers Robert Wood Johnson Medical School, New Brunswick, New Jersey 08901, USA; 24 Department of Laboratory Medicine and Pathology, Mayo Clinic, Rochester, Minnesota 55905, USA; 25 Department of Neurology, Mayo Clinic, Rochester, Minnesota 55905, USA; 26 Department of Neurology, Mayo Clinic, Jacksonville, Florida 32224, USA; 27 Department of Neurosciences, University of California, San Diego, La Jolla, California 92093, USA; 28 Center for Applied Genomics, Children’s Hospital of Philadelphia, Philadelphia, Pennsylvania 19104, USA

## Abstract

Corticobasal degeneration (CBD) is a neurodegenerative disorder affecting movement and cognition, definitively diagnosed only at autopsy. Here, we conduct a genome-wide association study (GWAS) in CBD cases (*n*=152) and 3,311 controls, and 67 CBD cases and 439 controls in a replication stage. Associations with meta-analysis were 17q21 at *MAPT* (*P*=1.42 × 10^−12^), 8p12 at *lnc-KIF13B-1*, a long non-coding RNA (rs643472; *P*=3.41 × 10^−8^), and 2p22 at *SOS1* (rs963731; *P*=1.76 × 10^−7^). Testing for association of CBD with top progressive supranuclear palsy (PSP) GWAS single-nucleotide polymorphisms (SNPs) identified associations at *MOBP* (3p22; rs1768208; *P*=2.07 × 10^−7^) and *MAPT* H1c (17q21; rs242557; *P*=7.91 × 10^−6^). We previously reported SNP/transcript level associations with rs8070723/*MAPT*, rs242557/*MAPT*, and rs1768208/*MOBP* and herein identified association with rs963731/*SOS1*. We identify new CBD susceptibility loci and show that CBD and PSP share a genetic risk factor other than *MAPT* at 3p22 *MOBP* (myelin-associated oligodendrocyte basic protein).

## Abstract (web summary)

Corticobasal degeneration is a rare neurodegenerative disorder that can only be definitively diagnosed by autopsy. Here, Kouri *et al.* conduct a genome-wide-association study and identify two genetic susceptibility loci 17q21 (*MAPT*) and 3p12 (*MOBP*), and a novel susceptibility locus at 8p12.

## Graphical abstract

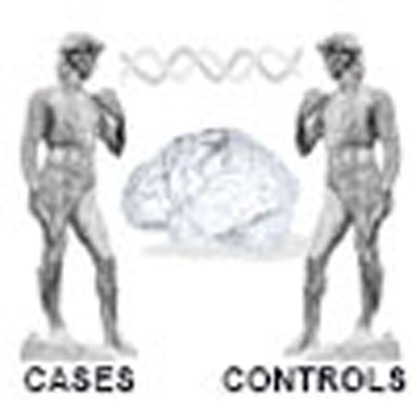

CBD is a late-onset neurodegenerative disorder that can only be diagnosed upon neuropathologic examination, which has impeded the ability to conduct genome-wide association studies (GWASs). Similar to progressive supranuclear palsy (PSP), Alzheimer’s disease (AD), and chronic traumatic encephalopathy, CBD is a tauopathy, having abnormal aggregates of microtubule-associated protein tau in the brain^1^. Neuropathologic diagnostic criteria for CBD are based on tau immunohistochemistry, requiring tau inclusions in neurons and glia, with tau astrocytic plaques, and extensive thread-like pathology in both grey matter and white matter^1^.

First described in 1968 by Rebeiz and colleagues, three CBD cases were identified to have a distinct movement disorder and neuropathologic profile^2^. These patients presented with what is now termed corticobasal syndrome (CBS) where patients can exhibit levodopa-unresponsive parkinsonism, asymmetric akinesia and rigidity, accompanied by ideomotor apraxia, dystonia, and myoclonus. Patients who present with the archetypal CBS, however, do not necessarily have CBD pathology upon neuropathologic examination. Furthermore, autopsy-confirmed CBD cases can often present with several different clinical syndromes^3^, which are frequently associated with other underlying neurodegenerative disorders such as PSP, AD, and frontotemporal dementia^4^. This phenotypic variability results in <50% of patients with CBS having CBD at autopsy^5^,^6^,^7^.

There are few reported families affected with CBD or PSP^8^,^9^,^10^, yet genetic association studies of PSP have repeatedly shown an increased risk for individuals carrying the H1 *MAPT* haplotype at chromosome 17q21 (refs 11, 12, 13, 14). An association of CBD with the H1 haplotype was first reported in a series of 18 cases (16 corticobasal syndrome patients and two autopsy-confirmed CBD cases)^15^ and subsequently confirmed in a larger autopsy series of 57 cases^16^. Recently, the first PSP GWAS^17^ discovered three non-*MAPT* susceptibility loci at *STX6*, *EIF2AK3*, and *MOBP*, raising the possibility that additional non-*MAPT* genetic risk factors or modifiers may also exist for CBD. Recognition of the lack of specificity of the corticobasal syndrome for CBD has promoted increasing interest among clinicians to have autopsy confirmation of patients thought to have CBD. Brain banks focusing on atypical parkinsonian disorders, such as that supported by the Foundation for PSP|CBD and Related Brain Diseases^18^, have facilitated accumulation of sufficient number of CBD cases to make identification of genetic contributions more feasible. Here, we report a two-stage GWAS in autopsy-proven CBD cases (*n*=152 discovery stage, *n*=67 replication stage) compared with controls (*n*=3,311 discovery stage, *n*=457 replication stage). The results confirm the association at *MAPT* and identify a novel susceptibility locus at 8p12 associated with CBD at the genome-wide significant level, as well as suggestive associations at 2p22 and 3p22. Importantly, we show that CBD and PSP share a genetic risk factor other than *MAPT*, at 3p22 *MOBP*. These findings highlight the genetic similarities and differences between CBD and PSP and suggest that the two disorders may, at least in part, share common disease processes.

## Results

### Discovery GWAS and replication

Genotype data from 152 autopsy-proven CBD cases and 3,311 controls (Table 1) were previously generated as part of the PSP GWAS^17^. The National Institute of Health Office of Rare Diseases Research criteria were used for making a neuropathologic diagnosis of CBD^1^. Discovery stage cases collected from multiple institutions (Supplementary Table 1) were genotyped by the Center for Applied Genomics at the Children’s Hospital of Philadelphia using Human 660 W-Quad Infinium BeadChips, and control samples were genotyped using the Illumina Human HapMap550 Infinium BeadChip. Replication stage samples included 67 autopsy-proven CBD cases (Mayo Clinic Florida Brain Bank, 6 CBD cases that failed quality control and 61 new CBD cases collected since the GWAS genotyping was performed) and 457 control individuals (mean age at blood draw=74 years) collected from Mayo Clinic Florida, all of which were clinically diagnosed as being free of any neurological disorder. For the replication stage, genotyping was performed on the 67 CBD cases using Taqman genotyping assays or Sanger sequencing and 30 CBD cases from the discovery stage as an internal genotyping control.

These data were subjected to accepted GWAS quality control measures. Individual samples were excluded with a genotype failure rate >2%, cryptic relatedness or duplicate samples based on identity by state. Genetic outliers were excluded on the basis of distance to the nearest-neighbour approach, and population substructure was assessed using multidimensional scaling (MDS) analysis with PLINK^19^ ( http://pngu.mgh.harvard.edu/~purcell/plink/; Supplementary Fig. 1). Quantile–quantile (QQ) plots and the genomic inflation factor (*λ*) show that the first principal component as a covariate in logistic regression analyses was sufficient to correct for population substructure based on a reduction of *λ* from the unadjusted (*λ*=1.06) to adjusted (*λ*=1.01; Supplementary Fig. 2).

**Figure 1:**
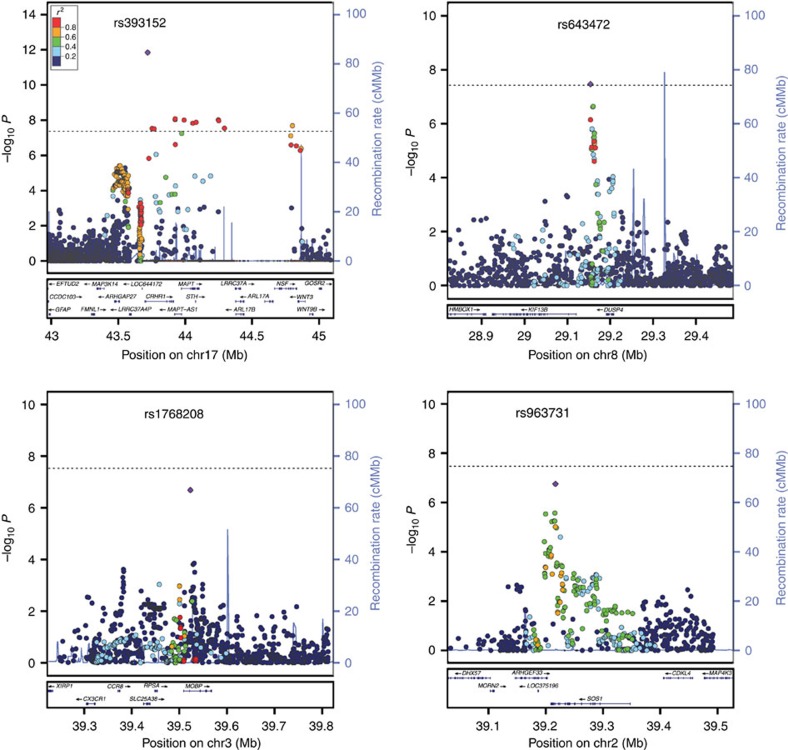
Regional association plots. (**a**–**d**) Top loci associated with CBD compared with controls in the Discovery Stage (152 CBD, 3,311 controls) and the Replication Stage (67 CBD, 457 controls). The most significant association is at 17q21, tagging the 1.2 Mb inversion containing *MAPT* (**a**). New susceptibility loci for CBD are located at 8p12 (**b**), 3p22 (**c**) and 2p22 (**d**). Genetic association testing was performed using conditional logistic regression analysis under an additive model and corrected for population substructure using the first principal component from multidimensional scaling analysis (MDS) as a covariate (*λ*=1.01). The *y* axis is −log_10_ *P* values and *x* axis is the genomic location of each SNP (circles) genotyped and imputed. Linkage disequilibrium coefficients were derived from hg19 (1,000 Genomes March 2012, European population) and local estimates of recombination rates are from HapMap samples (2008–03_rel22_B36; http://hapmap.ncbi.nlm.nih.gov). The random effects meta-analysis top SNP at each locus (purple diamonds).

For the discovery stage, we analysed association between disease and 533,898 single-nucleotide polymorphisms (SNPs) in 152 CBD cases and 3,311 control individuals by conditional logistic regression under an additive model using the first MDS principle component as a covariate using PLINK^19^. Given the neuropathologic, clinical, and genetic overlaps between CBD and PSP, we selected the top PSP GWAS SNPs at *MAPT* (the H1c subhaplotype-tagging SNP, rs242557), *MOBP*, *EIF2AK3*, and *STX6* to test for association with CBD. The discovery stage identified one genome-wide significant association at 17q21.31, which encompasses *MAPT* (rs393152, odds ratio (OR)=3.45, *P*=6.71 × 10^−9^) testing using conditional logistic regression analysis (Fig. 1a; Table 2) and 12 SNPs in LD with rs393152 (Supplementary Table 2). QQ plots including and excluding SNPs at 17q21 show that the *P* value distribution is consistent with the null, except for departures in the extreme tail (Supplementary Fig. 3). We also found nominal evidence for association testing using conditional logistic regression analysis at two novel susceptibility loci—rs643472 (OR=1.88, *P*=7.12 × 10^−7^), an intronic SNP in *lnc-KIF13B-1* (TCONS_00014956, a large intergenic non-coding RNA, or lincRNA) located between *KIF13B* and *DUSP4* on chromosome 8p12, and rs963731 (OR=2.46, *P*=2.04 × 10^−6^), an intronic SNP in *SOS1* on chromosome 2q22 (Supplementary Fig. 4). Testing for association using conditional logistic regression analysis with the top PSP GWAS SNPs identified rs1768208 (OR=1.65, *P*=3.86 × 10^−5^), an intronic SNP in *MOBP* (myelin-associated oligodendrocyte basic protein), and rs242557 (OR=1.48, *P*=1.20 × 10^−3^) at the *MAPT* locus to be associated with CBD. The top SNP at *EIF2AK3* (rs7571971) did not show an association with CBD (*P*=0.057; OR=1.27), but the OR was of similar effect size as observed for PSP (Supplementary Table 3).

In an independent cohort of 67 autopsy-proven CBD cases from the Mayo Clinic Florida Brain Bank and 457 control individuals, a replication stage was performed for a total of seven SNPs: the top five SNPs from the discovery stage (*P*<10^−5^) and two PSP GWAS SNPs that showed nominally significant association with CBD (rs1768208 and rs242557) (Table 2). Association testing for these seven SNPs was performed using conditional logistic regression under an additive model with age and sex as covariates. Using PLINK to perform random effects meta-analysis, or the weighted average of the effect sizes of the discovery and replication stages, showed genome-wide significant associations at *MAPT* (rs393152, *P*_meta_=1.4 × 10^−12^) (Fig. 1a) and *lnc-KIF13B-1* (rs643472, *P*_meta_=3.4 × 10^−8^; Table 2) (Fig. 1b). SNPs at *MOBP* (rs1768208*, P*_meta_=2.1 × 10^−7^) (Fig. 1c), *SOS1* (rs963731*, P*_meta_=1.8 × 10^−7^) (Fig. 1d) and *MAPT* H1c haplotype (rs242557, *P*_meta_=7.9 × 10^−6^) had suggestive evidence for association with CBD. Performing the same random effects meta-analysis while including the number of H1 alleles as a covariate, the association was lost (rs242557, OR=1.18, *P*_meta_=0.11). The MAFs for the top SNPs in younger controls of this study did not differ significantly from those of older controls (*N*=3,720) in three data sets from the National Institute of Health Database for Genotypes and Phenotypes (dbGaP; http://www.ncbi.nlm.nih.gov/gap; Supplementary Table 4). Furthermore, we repeated the discovery stage CBD GWAS with a different control data set, 1,986 controls from dbGaP data set NeuroGenetics Research Consortium (NGRC-PD; phs000196.v2.p1), analysed by logistic regression under an additive model and found similar association results (Supplementary Table 5).

Genotype imputation was performed to increase the resolution of the top susceptibility loci. CBD cases and controls were imputed to samples from the 1,000 Genomes Project (May 2011, European reference population)^20^. Phasing was performed with SHAPEIT v2.r790 (ref. 21; https://mathgen.stats.ox.ac.uk/genetics_software/shapeit/shapeit.html) and genotype imputation was performed with IMPUTE2 v2.3.1 (ref. 22; https://mathgen.stats.ox.ac.uk/impute/impute_v2.html). Genomic coordinates for imputation were selected at the most significant SNPs and their surrounding gene(s). Testing for association of CBD with imputed genotypes did not improve association results.

Top susceptibility loci were further examined with HaploRegv2 ( http://www.broadinstitute.org/mammals/haploreg/documentation_v2.html), a tool for chromatin profiling, which allows one to explore the impact of non-coding variants, such as those identified in GWASs, by using *in silico* analysis coupled with experiments to validate biochemical and functional activators and repressors^23^,^24^. Results show that rs643472 is located in an enhancer region in multiple cell lines and alters three transcription factor motifs, rs963731 at 2p22 alters 18 regulatory motifs, and rs1768208 at 3p22 is in high LD (*R*^2^=0.89–0.98) with five SNPs that are located in enhancer regions in several brain regions (Supplementary Table 6 and Supplementary Fig. 5). Two of the five SNPs (rs631312 and rs1768190) were further genotyped in CBD (*n*=133) and controls (*n*=457), and association testing showed similar results with CBD (OR=1.89 (CI=1.30–2.75), *P*=8.77 × 10^−4^; OR=1.91 (CI=1.31–2.77), *P*=7.24 × 10^−4^, respectively) to rs1768208 (Table 3).

### Expression quantitative trait locus analysis

To further explore potential biological mechanisms, RNA expression quantitative trait loci (eQTL) analysis for the CBD risk loci were interrogated in our expression GWAS (eGWAS)^25^ of ∼400 samples from cerebellum and temporal cortex from neurodegenerative disorders (Supplementary Table 7) using the Illumina Whole-Genome DASL assay. Expression changes are a possible mechanism of disease-associated functional variants, but other mechanisms also need to be investigated in future studies. The eGWAS data were queried for the top SNPs associated with CBD (rs393152, rs963731, rs1768208, and rs242557). The eGWAS genotypes did not contain rs643472 (*KIF13B*/*DUSP4* locus) or any SNPs in LD at *R*^2^>0.5 with rs643472; and the call rates for the two *DUSP4* expression probes did not pass quality control, precluding eQTL analysis with *DUSP4* expression. SNPs were tested for association with transcript levels in the eGWAS cohort by multivariable linear regression using an additive model, with the minor allele dosage (0, 1 or 2) as the independent variable, and APOE ε4 dosage (0, 1 or 2), age at death, gender, PCR plate, RIN, and RIN-RIN_mean_ as biological and technical covariates. The sample set was analysed as a single cohort for maximum statistical power to detect associations, while including a covariate for AD diagnosis. As we previously reported^25^, there were significant SNP/transcript associations with *MAPT*/rs8070723 (cerebellum: *P*=7.02 × 10^−69^; temporal cortex: *P*=8.61 × 10^−44^) and *MOBP*/rs1768208 (cerebellum: *P*=1.71 × 10^−7^; temporal cortex: 1.57 × 10^−6^). We also detected significant associations with *SOS1*/rs963731 (cerebellum: *P*=4.61 × 10^−4^; temporal cortex: *P*=2.80 × 10^−6^) upon eQTL analysis (Table 4). Beta values for SNP/transcript associations showed increased expression levels with each copy of the risk allele.

To determine whether the novel genome-wide significant association at 8p12 (rs643472, an intronic SNP in *lnc-KIF13B-1*) genotype correlates with differential expression levels, we measured *lnc-KIF13B-1* expression in RNA from superior frontal cortex of 22 CBD and 20 normal cases using the mirVana Paris RNA isolation kit and quantitative PCR (qPCR) by SYBR Green. All qPCR reactions were technically replicated. Two different qPCR assays targeting TCONS_00014956 were designed, one targeting exon 1 (primer set A) and the other for exon 2 (primer set B), which showed good correlation upon comparison of expression levels using the two different primer sets (*R*^2^=0.97). This identified a statistical trend for greater *lnc-KIF13B-1* expression with the minor risk allele (Supplementary Fig. 6).

## Discussion

This first CBD GWAS on 152 autopsy-proven CBD patients and 3,311 control individuals has a clear limitation in terms of sample size. Given that CBD is a rare disorder and that it can only be diagnosed upon neuropathologic examination, this was the largest possible cohort at the time of genotyping. To control the false-positive rate due to the small sample size, a stringent significance threshold (*P*<10^−5^) was applied in the discovery stage to select SNPs for genotyping in the replication stage. This proved to be a useful approach because the random effects meta-analysis identified two associations that did not replicate (Table 2). The disadvantage of applying stringent criteria is the possibility of false-negative associations, as evidenced from the power analysis, which show low power to detect genome-wide associations of variants with modest effect sizes (Supplementary Tables 8 and 9).

Both CBD and PSP now have confirmed genome-wide significant associations at the *MAPT* locus. In addition, CBD was also associated with rs242557 (*P*=7.91 × 10^−6^), a SNP tagging the H1c subhaplotype at 17q21, shown to be associated with PSP and located in a regulatory region influencing *MAPT* expression^26^. In support of the *MOBP* locus being a shared genetic risk factor between CBD and PSP, CBD cases have an even greater OR estimate compared with PSP (MAF_CBD_=0.40; OR=1.71; MAF_PSP_=0.36; OR=1.39; ref. 17). Importantly, this is the first non-*MAPT* genetic risk factor shared between CBD and PSP. Yamamoto *et al.*^27^ described *MOBP* as one of the most abundant oligodendrocyte-expressing proteins, similar to myelin basic protein, with the main difference being that *MOBP* is only expressed in myelin of the central nervous system. The results of the current study and those of the PSP GWAS indicate an overlap in genetic risk factors for PSP and CBD, but it appears that there is additional genetic variation that differs between the two disorders. Additional analyses of the novel susceptibility loci at chromosomes 8p12 and 2p22 have the potential to advance our understanding of CBD. The chromosome 8p12 locus contains three candidate genes—*lnc-KIF13B-1* (TCONS_00014956, a large intergenic non-coding RNA or lincRNA); kinesin family member 13B (*KIF13B*); and dual-specificity protein phosphatase 4 (*DUSP4*), also known as MAP kinase phosphatase-2 (MKP-2)). Although the *lnc-KIF13B-1* expression levels were not significantly different between CBD and normals, there was a trend for greater expression with the minor risk allele. KIF13B is a plus end-directed microtubule motor protein highly expressed in the brain, and is involved in synaptic vesicle trafficking along microtubules, neurite extension, and caveolin-dependent endocytosis^28^,^29^,^30^. Of interest is that one of the PSP risk variants is in the gene *STX6* and is a SNARE-class protein that regulates vesicle membrane fusion, which raises the possibility that dysfunction of vesicular trafficking may be a common disease mechanism between CBD and PSP.

*DUSP4* acts through tyrosine and threonine-directed dephosphorylation^31^. Because tau phosphorylation plays a critical role in tauopathies^32^, it will be interesting if *DUSP4* is responsible for the genetic association at 8p12. Indeed, in Alzheimer's disease brain tissue there is decreased enzymatic activity of tau phosphatases such as PP-2A and PP-1 (ref. 33), indicating that a potential role for variants in *DUSP4* in CBD pathogenesis would be through aberrant tau phosphorylation. Furthermore, the chromosome 2p22 locus contains son of sevenless homologue 1 (*SOS1*), a guanine nucleotide exchange factor for Ras that has a catalytic region known as the cdc25 domain due to sequence homology to CDC25, a dual-specificity phosphatase^34^. Taken together, the novel susceptibility loci for CBD may link common genetic variation to aberrant tau phosphorylation.

In conclusion, this first CBD GWAS identified *MAPT* and *MOBP* as shared genetic risk factors between CBD and PSP. Given the significant white matter and oligodendrocyte pathology in these primary tauopathies, the genetic association with *MOBP* warrants functional characterization to determine its role in CBD and PSP.

## Methods

### Samples

The discovery stage cohort was comprised of 152 autopsy-proven CBD cases collected from eight institutions (Table 1 and Supplementary Table 1). Discovery stage controls were recruited from the Children’s Hospital of Philadelphia Health Care Network, and although these controls are not age matched to the cases, the justification for using this cohort is that they were all genotyped at the same center using the same protocol as the CBD cases. Furthermore, because CBD is such a rare disorder with an estimated prevalence of 4.9–7.3 cases per 100,000 individuals^35^, the chance of any of the young controls developing CBD at a later age is negligible. As described in the PSP GWAS^17^, to ensure significant associations were not confounded by using young controls, allele frequencies for the top associated SNPs were compared and found to be similar to older controls (*N*=3,720) from three data sets downloaded from the National Institute of Health (NIH) Database for Genotypes and Phenotypes (dbGaP).

DNA was extracted from the brain tissue of CBD patients and from the blood samples of controls. Brain autopsies were obtained after consent of the legal next-of-kin and are considered exempt from human subject research. Written informed consent was obtained from control individuals as approved by The Institutional Review Board (University of Pennsylvania) and Mayo Clinic Institutional Review Board.

Power analyses were estimated for the Discovery Stage and Replication Stage for various minor allele frequencies and odds ratios with 1,000 simulations of each scenario (Supplementary Tables 8 and 9). The power assessment assumes that genotype frequencies are in Hardy–Weinberg equilibrium in CBD cases and control individuals separately and that association testing is performed using logistic regression under an additive model.

### Quality control

Quality control of genotyping data was performed at the individual level and then at the SNP level. For quality control, 10 individuals were genotyped in duplicate. Exclusion criteria for individual samples included high genotype failure rate (six individuals were removed because of a genotype failure rate >2%) and cryptic relatedness. Genetic outliers were excluded from further analyses on the basis of identity by state and distance to the nearest-neighbour analysis was conducted in PLINK^19^ (five CBD cases and 341 controls were removed on the basis of *Z*-score distributions for the first to the fifth neighbour, *Z*-score<−2). Gender inconsistencies were assessed by chromosome X genotypes, which excluded six individuals on the basis of observed and expected gender. Exclusion criteria for markers included minor allele frequency (24,183 SNPs were removed because of minor allele frequency <1%), deviation from Hardy–Weinberg equilibrium in controls (1,098 SNPs gave a Hardy–Weinberg expectations test, *P* value ≤10^−7^), and high genotype failure rates (3,199 SNPs were removed because of genotype failure rates >2%).

### Population substructure

MDS was applied to a pruned data set (∼140,000 markers) using PLINK. Scatter plots for the first two principle components were generated using R (Supplementary Fig. 1a). Analysis of population stratification showed that the first principle component was sufficient to reduce the genomic inflation factor (*λ*) from 1.06 to 1.01, as illustrated by the quantile–quantile (QQ) plots of observed versus expected *P* values. Using the first two principal components resulted in *λ*=1.02 (Supplementary Fig. 2b); thus, only the first MDS principal component was used as a covariate for all subsequent analyses.

### Association analysis

CBD versus control association was tested by conditional logistic regression under an additive model using the first MDS principle component as a covariate. We also tested for association from a subset of samples from stage 1, excluding outliers based on population substructure (Supplementary Fig. 1b), which yielded similar results. For rs242557, additional association testing was performed, conditioning on *MAPT* haplotype (that is, using 0, 1 or 2 as a covariate for the number of H1 alleles per individual). Linkage disequilibrium values were derived from HapMap 3 release 27.

CBD cases and controls were imputed up to the 1000 Genomes European reference^20^, May 2011 release (phase 1, version 3; hg19). SHAPEIT v2.r790 (ref. 21) was used for phasing and IMPUTE2 v2.3.1 (ref. 22) was used for genotype imputation. Imputed variants with MAF <0.01 or dosage *R*^2^<0.3 were excluded, and all downstream analyses were based on the most likely genotype^36^. Regional genome-wide association plots were created with LocusZoom^37^ ( http://csg.sph.umich.edu/locuszoom/). HaploRegv2 (Broad Institute) uses linkage disequilibrium information from 1000 Genomes Project, SNPs (based on dbSNP 137), motif instances (based on PWMs discovered from ENCODE experiments), and enhancer annotations (adding 90 cell types from the Roadmap Epigenome Mapping Consortium)^23^,^24^.

### eQTL analysis

These data were generated and previously reported in a brain expression GWAS (eGWAS) by Zou *et al.*^25^, where the detailed mRNA extraction and quality assessment using RNAqueous kit (Ambion, Grand Island, NY) and RNA 6,000 Nano Chip (Agilent, Santa Clara, CA), Whole Genome DASL assay (Illumina, San Diego, CA), and data quality control methods can be found. This eGWAS cohort included 374 cerebellar and 399 temporal cortex mRNA samples from the Mayo Clinic Florida Brain Bank. Neuropathologic diagnoses for the cohort include Alzheimer’s disease, PSP, CBD, Lewy body disease, frontotemporal lobar degeneration, and other diagnoses (Supplementary Table 7). The Bonferroni *P* value threshold for SNP/transcript association significance was *P*<1.56 × 10^−3^ (four SNPs and eight probes).

### RNA preparation for *lnc-KIF13B-1* expression studies

Superior frontal cortex tissue was isolated from CBD cases and normal controls from the Mayo Clinic Florida brain bank and matched for age at death and gender. Total RNA from brain tissue samples was extracted with acid–phenol: chloroform chemistry using mirVana PARIS isolation kit (Ambion). Total RNA concentration was determined with a NanoDrop ND-1,000 spectrophotometer (NanoDrop, Wilmington, DE). RNA quality control was performed using a 2,100 Bioanalyzer (Agilent, Santa Clara, CA) to measure RNA integrity number (RIN) and only samples with an RNA integrity value >5 were included in this study. The mean RIN in frontal cortex for 22 CBD cases (5.6±0.38) were not different from 19 controls (6.4±0.77).

### Real-time quantitative PCR for lincRNA

Five hundred nanograms of total RNA from the human superior frontal of CBD patients (*n*=22) and normal controls (*n*=19) were reverse transcribed with High Capacity cDNA Reverse Transcription kit (Applied Biosystems, Carlsbad, CA). Quantitative PCR (qPCR) was performed using Fast SYBR Green PCR Master Mix (Applied Biosystems) and StepOnePlus (Applied Biosystems) according to the company’s suggested protocol and thermal cycling programme. A final dissociation step was performed at the end of the qPCR assay to evaluate the specificity of amplification. All qPCR reactions were technically replicated. Two different qPCR assays targeting TCONS_00014956 were designed, one targeting exon 1 (Primer set A) and the other for exon 2 (Primer set B). All the primers were purchased from Sigma Life Science (Sigma, St Louis, MO) with the sequences shown in Supplementary Fig. 6. The relative levels of lincRNA were calculated by comparative Ct method using StepOne Software v2.3 (Applied Biosystems, Grand Island, NY) and GeneEx 5.3.2 (Multid Analyses, Göteborg, Sweden). GAPDH was used as a normalization control. Statistical analysis was performed using GraphPadPrism v5.04 (GraphPad, La Jolla, CA).

## Additional information

**How to cite this article**: Kouri, N. *et al.* Genome-wide association study of corticobasal degeneration identifies risk variants shared with progressive supranuclear palsy. *Nat. Commun.* 6:7247 doi: 10.1038/ncomms8247 (2015).

## Supplementary Material

Supplementary Information Supplementary Figures 1-6 and Supplementary Tables 1-9

## Figures and Tables

**Table 1:** Description of samples.

	**Female**			**Age onset/blood draw**			**Age at death**			**Disease duration**		
**Cohort**	**Total**	**%**	***n*** *	**Mean age**	**Range (s.d.)**	***n***	**Mean age**	**Range (s.d.)**	***n***	**Mean duration** †	**Range (s.d.)**	***n***
Discovery stage‡												
CBD cases	152	50	150	64	33–80 (8.6)	103	70	44–91 (9.2)	144	6	2–14 (2.4)	103
Replication stage												
CBD cases	67	48	67	64	40–83 (7.9)	67	70	54–96 (7.8)	67	6	2–14 (2.5)	61
Controls	457	54	457	74	24–97 (13.1)	457	—	—	—	—	—	—

^*^n is the number of samples with available data. Values of n for each type of analysis do not add up to the total samples used because of missing values.

^†^Duration in years.

^‡^Controls (*n*=3,311) were young healthy subjects recruited from the Children’s Hospital of Philadelphia Health Care Network (see Online Methods for details).

**Table 2:** Results from discovery stage, replication stage and meta-analysis.

				**MAF**			
**SNP**	**Chr**	**Gene(s)**	**Stage** *	**Cases**	**Controls**	**OR (95% CI)**	***P***
rs393152†	17q21	*MAPT*	Discovery	0.079	0.24	3.45 (2.29–5.34)	6.71 × 10^−9^
		(H1/H2)	Replication	0.068	0.24	4.37 (2.16–8.83)	4.12 × 10^−5^
			Meta-analysis			3.70	1.42 × 10^−12^
rs643472	8p12	*lnc-KIF13B-1*	Discovery	0.35	0.23	1.88 (1.46–2.40)	7.12 × 10^−7^
			Replication	0.32	0.22	1.67 (1.11–2.52)	0.014
			Meta-analysis			1.82	3.41 × 10^−8^
rs963731	2p22	*SOS1*	Discovery	0.12	0.054	2.46 (1.70–3.57)	2.04 × 10^−6^
			Replication	0.09	0.049	2.21 (1.09–4.50)	0.029
			Meta-analysis			2.41	1.76 × 10^−7^
rs1768208	3p22	*MOBP*	Discovery	0.39	0.29	1.65 (1.30–2.09)	3.86 × 10^−5^
			Replication	0.39	0.25	1.89 (1.28–2.77)	1.30 × 10^−3^
			Meta-analysis			1.71	2.07 × 10^−7^
rs242557	17q21	*MAPT*	Discovery	0.45	0.35	1.48 (1.17–1.88)	1.50 × 10^−3^
		(H1c)	Replication	0.48	0.36	1.77 (1.25–2.52)	1.50 × 10^−3^
			Meta-analysis			1.57	7.91 × 10^−6^
rs1860743	7q36	*PRKAG2*	Discovery	0.20	0.11	2.05 (1.52–2.78)	3.46 × 10^−6^
			Replication	0.09	0.13	0.71 (0.39–1.31)	0.27
			Meta-analysis			1.25	0.67
rs875125	21q22	*TSPEAR*	Discovery	0.17	0.10	2.02 (1.48–2.75)	7.92 × 10^−6^
			Replication	0.05	0.08	0.65 (0.29–1.46)	0.29
			Meta-analysis			1.22	0.73

^*^Discovery stage (152 CBD, 3,311 controls); Replication stage (67 CBD, 457 controls). The discovery and replication stages were analysed by logistic regression under an additive model. The discovery stage analysis was adjusted for the first multidimensional scaling principle component, and the replication stage was adjusted for age and sex.

^†^The OR for rs393152 is referencing the risk associated with the major allele (*MAPT* H1 haplotype). The protective allele (*MAPT* H2 haplotype) has an OR (95% CI) of 0.29 (0.19–0.44) in the discovery stage and 0.23 (0.11–0.46) in the replication stage. Genome-wide significantceis defined as variants associated with *P*<5 × 10^−8^. Chr, chromosome; CI, confidence interval; lnc, long non-coding (RNA); MAF, minor allele frequency; OR, odds ratio.

**Table 3:** Association with Mayo CBD cohort and SNPs at *MOBP* predicted to be in regulatory enhancer regions.

**SNP**	**Gene**	**CBD MAF**	**Control MAF**	**LD** ***R***^**2**^ rs1768208*	**OR (95% CI)**	***P***
rs631312	*MOBP* (Upstream)	0.39	0.25	0.84	1.89 (1.30–2.75)	8.77 × 10^−4^
rs1768190	*MOBP* (Intron 1)	0.40	0.26	0.78	1.91 (1.31–2.77)	7.24 × 10^−4^
rs1768208	*MOBP* (Intron 2)	0.40	0.29	—	1.89 (1.28–2.77)	1.27 × 10^−3^

Mayo CBD cohort is composed of 133 autopsy-proven CBD cases and were tested for association with 457 controls from the replication stage. Association testing was performed using logistic regression under an additive model, with age and sex as covariates.

^*^Linkage disequilibrium (LD) values from HapMap release 27 with top *MOBP* SNP associated with CBD, rs1768208 (Meta-analysis results, OR=1.71, *P*_meta_=2.07 × 10^−7^).

**Table 4:** CBD GWAS SNP/transcript associations in cerebellar and temporal cortex human tissue samples.

				**Cerebellum**		**Temporal cortex**	
**SNP**	**Chr**	**Gene**	**Probe**	**Beta**	***P***	**Beta**	***P***
rs963731	2	*SOS1*	ILMN_1767135	0.154	4.61 × 10^−4^	0.178	2.80 × 10^−6^
rs1768208	3	*MOBP*	ILMN_2298464	0.363	1.71 × 10^−7^	0.325	1.57 × 10^−6^
rs1768208	3	*MOBP*	ILMN_2414962	0.243	1.17 × 10^−4^	0.180	1.76 × 10^−5^
rs1768208	3	*MOBP*	ILMN_1768947	0.143	7.57 × 10^−3^	0.107	3.03 × 10^−3^
rs1768208	3	*MOBP*	ILMN_1750271	0.074	0.021	0.046	0.016
rs242557	17	*MAPT*	ILMN_1710903	0.183	8.80 × 10^−13^	0.181	1.10 × 10^−8^
rs242557	17	*MAPT*	ILMN_2298727	0.071	9.78 × 10^−3^	0.041	0.11
rs242557	17	*MAPT*	ILMN_2310814	0.002	0.83	−0.026	0.22
rs8070723	17	*MAPT*	ILMN_1710903	−0.473	7.02 × 10^−69^	−0.498	8.61 × 10^−44^
rs8070723	17	*MAPT*	ILMN_2298727	−0.173	3.36 × 10^−7^	−0.137	9.03 × 10^−4^
rs8070723	17	*MAPT*	ILMN_2310814	−0.007	0.58	0.018	0.37

Top CBD GWAS SNPs were tested for transcript associations in 374 human cerebellum and 399 temporal cortex samples. Linear regression was employed using an additive model, with the minor allele dosage (0, 1, 2) as the independent variable, and APOE ε4 dosage (0, 1, 2), age at death, gender, PCR plate, RIN, (RIN-RINmean) as biological and technical covariates^14^.
