# Supplementary material for: Genome-wide association study of corticobasal degeneration identifies risk variants shared with progressive supranuclear palsy
Source: Nat Commun. 2015 Jun 16;6:7247. doi: 10.1038/ncomms8247 (PMC4469997; doi:10.1038/ncomms8247)
Supplement: Supplementary Information — Supplementary Figures 1-6 and Supplementary Tables 1-9 [file ncomms8247-s1.pdf]

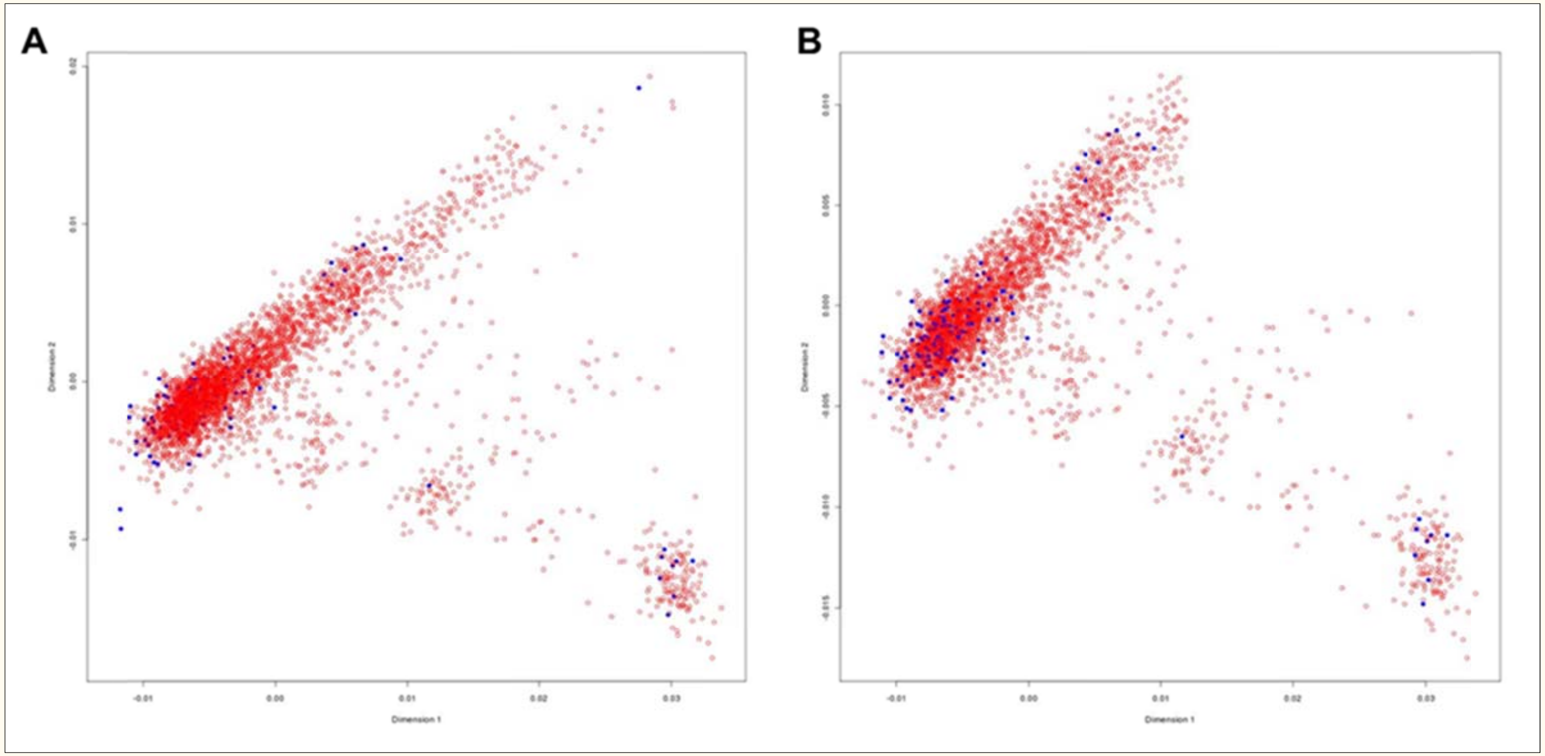

Supplementary Figure 1. Multidimensional scaling plots for CBD cases and control individuals

Multidimensional scaling (MDS) components were generated on a pruned dataset of ~140,000 markers using PLINK. The x-axis represents the first principle component and the y-axis is the second principle component. CBD cases (blue solid circles, N = 152); Children's Hospital of Philadelphia control individuals (red open circles, N = 3,311).

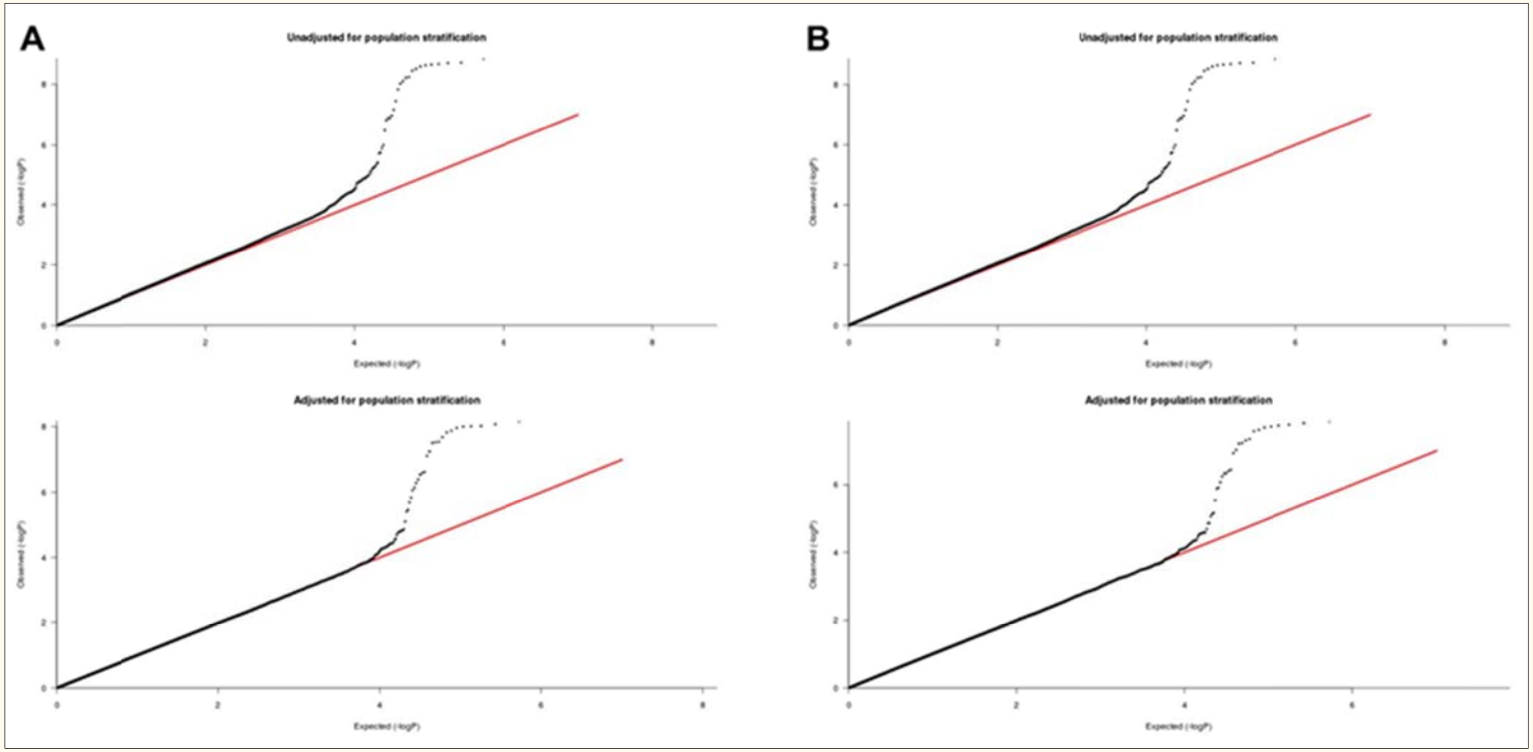

Supplementary Figure 2. Quantile-quantile plots in CBD genome-wide scan

Observed versus expected *P* values for the unadjusted logistic regression analysis (top panels) compared to an adjusted analysis using the first MDS generated principle component as a covariate in a logistic regression model for discovery stage samples (152 CBD, 3,311 controls, and a ~140,000 marker pruned dataset) (**A**). The principle components generated by MDS and the individual SNPs in the study were used to calculate the genomic inflation factor (λ). Based on the value of λ, the first component from the MDS analysis in the logistic regression model was sufficient to control for population substructure. Unadjusted for population substructure (λ=1.06) and adjusting for the first MDS generated principle component (λ=1.01). (**B**) Genomic inflation factor λ=1.06 for unadjusted and λ=1.02 when adjusting for the first and second MDS

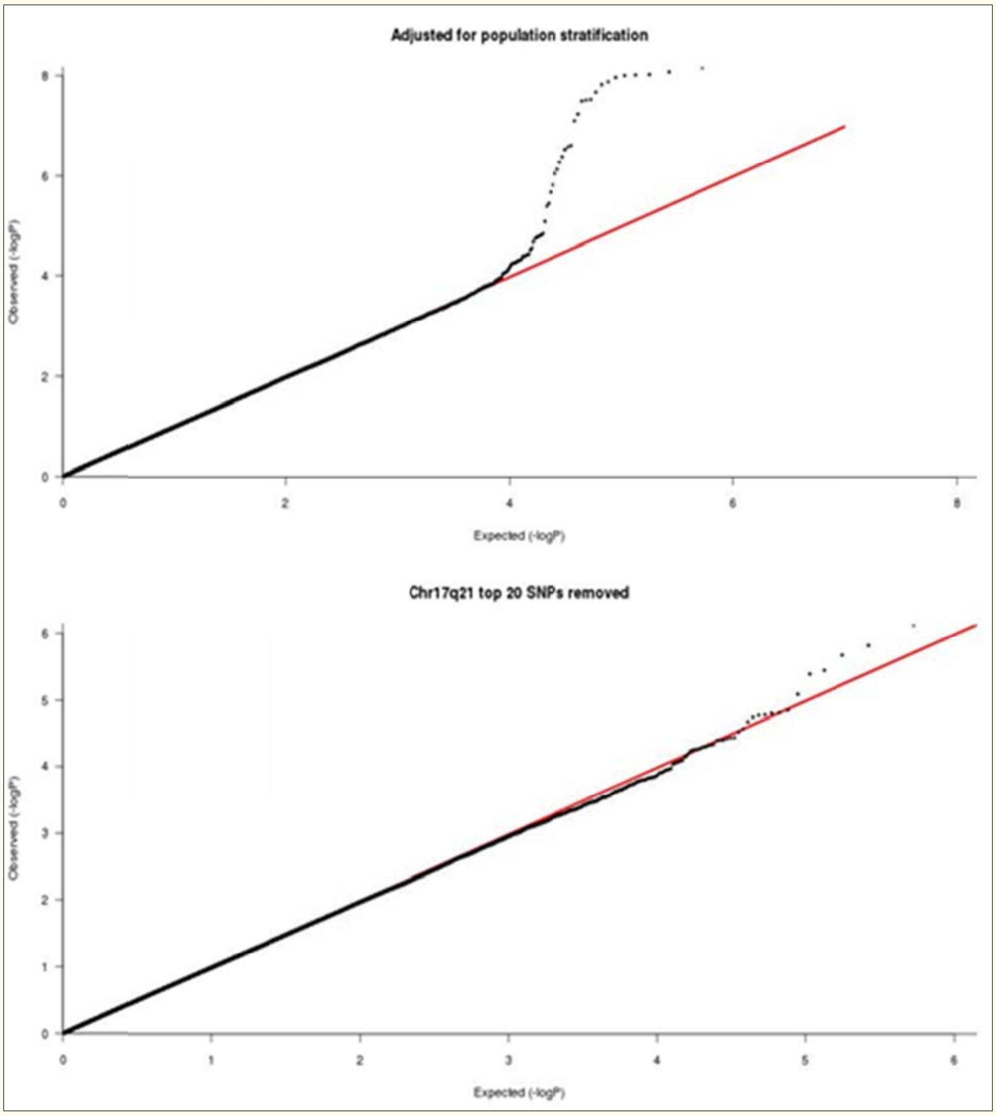

Supplementary Figure 3. Quantile-quantile (QQ) plots in CBD genome-wide scan excluding all SNPs at 17q21.31 locus

Departures in the extreme tail of the distribution of test statistics are due to regions with a strong signal for association by conditional logistic regression, additive model, and the first principle component (multidimensional scaling) as a covariate using PLINK for discovery stage samples (152 CBD, 3,311 controls, and a ~140,000 marker pruned dataset). The strongest association was with rs393152 ($P = 6.7 \times 10^{-9}$) at chromosome 17q21.31 locus, and 14 additional SNPs meet the threshold for genome-wide significance ($P < 5 \times 10^{-8}$). QQ plot of the results of the trend test (top) compared to the QQ plot excluding all SNPs located at the chromosome 17q21.31 locus (bottom), show that departures in the extreme tail of the distribution of test statistics are due to regions with a strong signal for association.

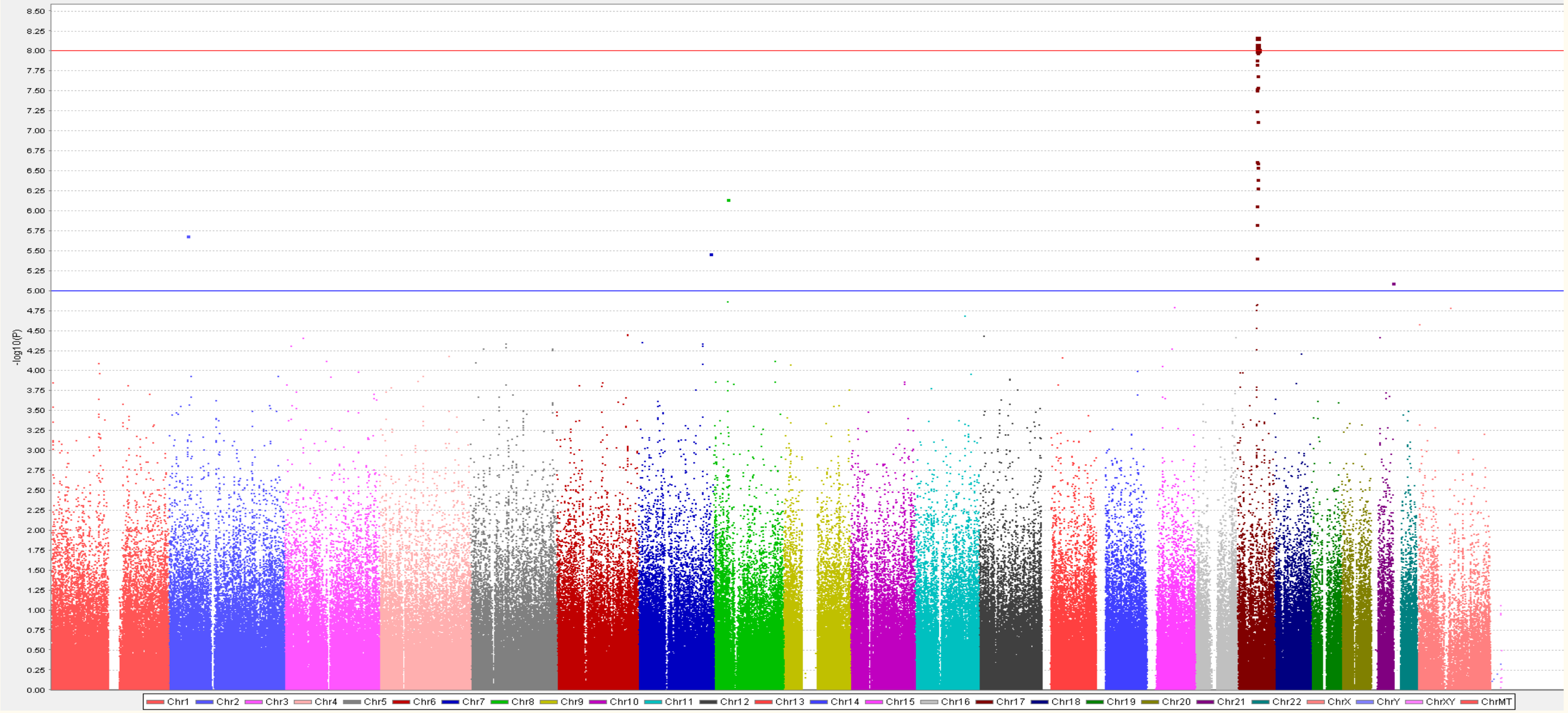

Supplementary Figure 4. Manhattan plot for CBD GWAS Discovery Stage.

For the discovery stage, we analyzed association between disease and 533,898 SNPs in 152 CBD cases and 3,311 control individuals by conditional logistic regression under an additive model using the first multidimensional scaling principle component as a covariate. Chromosomes are delineated by different colors, as labeled on the x axis. The y axis shows $-\log_{10} P$ values.

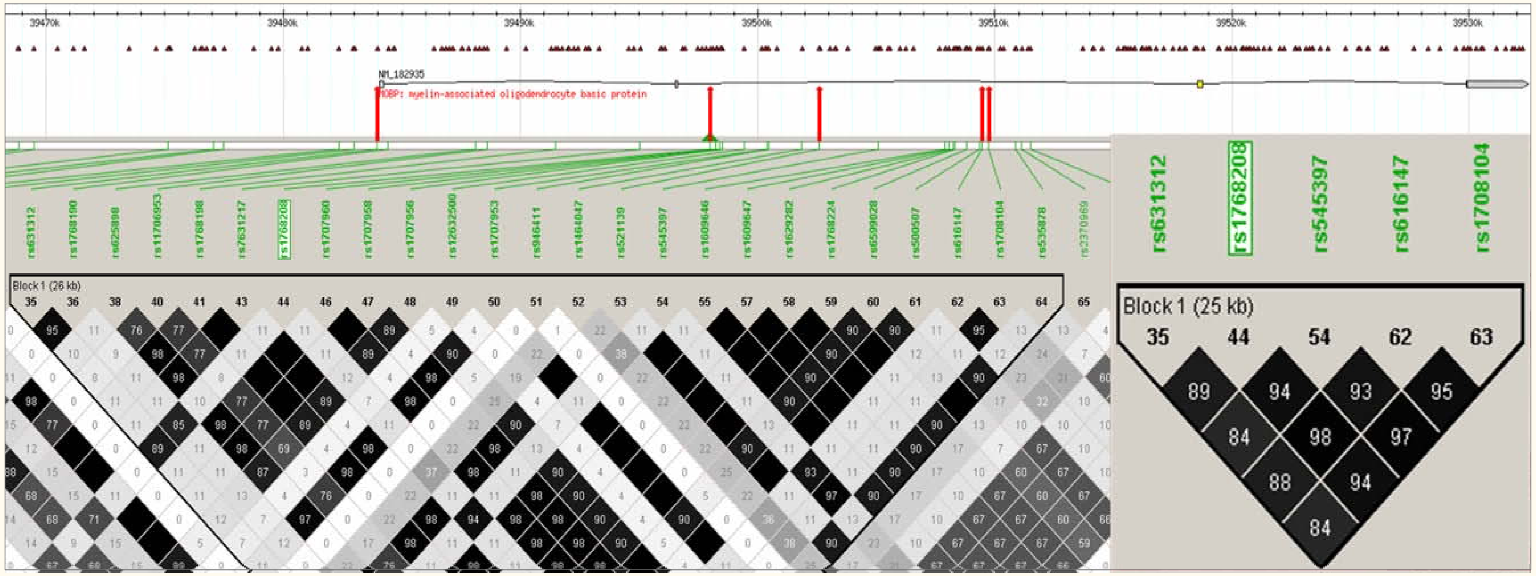

Supplementary Figure 5. Linkage disequilibrium structure at 3p22 (rs1768208).

Location of the intronic SNPs in *MOBP*, a new CBD susceptibility locus. Top SNPs associated with CBD are in strong LD with rs1768208 (rs631312, rs545397, rs1513219, rs616147, rs1708104), all of which are located in enhancer regions in several brain regions (please also refer to Supplementary Table 7 for hyperlinks to HaploReg v2 data for each SNP). LD values (R^2) are from HapMap 3 release 27 (CEU+TSI). Inset is the LD structure at the top SNPs associated with CBD in this region.

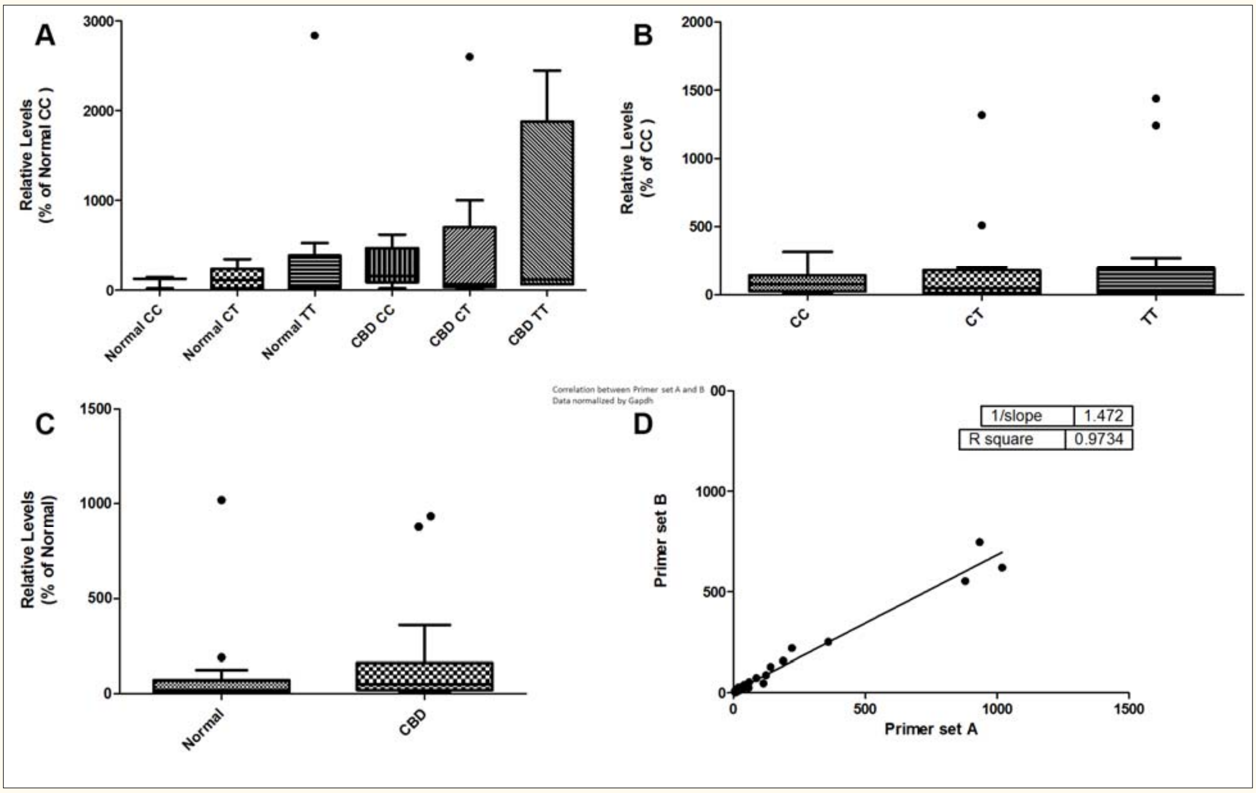

Primer ID	Length	Tm	GC%	Sequence	Position (GRCh37/hg19)
Primer A Forward	23	59	48	CCTCATCTGTCTGAAACCTTGCT	chr8:29,150,113-29,150,135
Primer A Reverse	21	58	52	TGCAGAGACTCCAGGGTCATT	chr8:29,150,065-29,150,085
Primer B Forward	20	59	55	TCCCTCCTTGCCTTGAGTCA	chr8:29,143,472-29,143,491
Primer B Reverse	23	58	48	CCTGCTTCTACAATGGGACTTGT	chr8:29,143,418-29,143,440

Supplementary Figure 6. Expression studies of *lnc-KIF13B-1* in CBD and Normal brain samples.

Total RNA was isolated from superior frontal cortex tissue from 23 CBD cases and 19 normal controls. Quantitative real time PCR was performed using SYBR green and expression levels were compared between normal and CBD stratified by genotype (**A**), by genotype only (**B**), and comparing all normal to all CBD (**C**). Two different primer sets were used, one targeting exon1 (primer set A) and the other targeting exon 2 of *lnc-KIF13B-1* (primer set B), which show good correlation (R^2 = 0.97) (**D**). Relative quantification values were calculated by normalizing to *GAPDH*. Primer sequences and features are listed.

Supplementary Table 1. Sample source of CBD cases with autopsy confirmation from Discovery Stage		
**Source**	**Number Genotyped**	**Number in Analysis**
Mayo Clinic	78	72
University of Pennsylvania	30	30
McLean Brain Bank	8	8
Indiana University School of Medicine	12	11
Brain Net Europe, Munich	8	7
NY Brain Bank, Columbia University	6	6
U Texas Southwestern Medical Center	6	6
Emory University	13	12
TOTAL	161	152

Supplementary Table 2. Twelve additional SNPs at Chr. 17q21.31 with genome-wide significant associations with CBD

CHR	SNP	BP[a]	Gene/feature	CBD MAF	Control MAF	OR	L95	U95	P
17q21	rs393152	41074926	*CRHR1*/intron	0.079	0.24	0.29	0.19	0.44	6.71E-09
17q21	rs12373139	41279910	*IMP5*/missense	0.079	0.24	0.29	0.19	0.44	8.27E-09
17q21	rs12185268	41279463	*IMP5*/missense	0.079	0.24	0.29	0.19	0.44	9.28E-09
17q21	rs2532274	41602941	*KANSL1*/intron	0.086	0.24	0.30	0.20	0.45	9.52E-09
17q21	rs17563986	41347100	*MAPT*/intron	0.079	0.24	0.29	0.19	0.44	9.78E-09
17q21	rs2532269	41605885	*KANSL1*/intron	0.082	0.24	0.30	0.20	0.45	1.07E-08
17q21	rs8070723	41436901	*MAPT*/intron	0.082	0.24	0.30	0.20	0.45	1.30E-08
17q21	rs1981997	41412603	*MAPT*/intron	0.082	0.24	0.30	0.20	0.46	1.47E-08
17q21	rs7224296	42155230	*NSF*/intron	0.13	0.30	0.38	0.28	0.54	2.05E-08
17q21	rs2668692	41648797	*KANSL1*/intron	0.083	0.23	0.31	0.20	0.47	2.85E-08
17q21	rs1635291	41107696	*CRHR1*/intron	0.11	0.27	0.35	0.24	0.51	2.94E-08
17q21	rs7215239	41123556	*CRHR1*/intron	0.11	0.27	0.35	0.25	0.51	3.08E-08

[a]All physical positions are from the Human Reference Genome Release 36. The odds ratios (ORs) are based on the minor protective allele (H2 *MAPT* haplotype). BP base-pair position, L95 Lower 95% confidence interval, U95 Upper 95% confidence interval

**Supplementary Table 3. Results for CBD association with top PSP GWAS SNPs**									
**CHR**	**SNP**	**Gene/feature**	**CBD MAF**	**Control MAF**	**PSP MAF**	**OR**	**L95**	**U95**	**P**
17q21	rs8070723	*MAPT*/intron	0.08	0.24	0.05	3.34	2.21	5.07	1.30E-08
3p22	rs1768208	*MOBP*/intron	0.39	0.29	0.36	1.65	1.30	2.09	3.86E-05
17q21	rs242557	*MAPT*/intron	0.45	0.35	0.53	1.48	1.17	1.88	1.20E-03
2p11	rs7571971	*EIF2AK3*/intron	0.31	0.26	0.31	1.27	0.99	1.64	0.057
1q25	rs1411478	*STX6*/intron	0.46	0.42	0.50	1.20	0.95	1.50	0.13

CBD (N = 152) versus discovery stage controls (N = 3,311) association with the top PSP GWAS SNPs was performed using logistic regression under an additive model and the first principle component (multidimensional scaling) as a covariate using PLINK. The odds ratios (ORs) are based on the major risk allele (H1 *MAPT* haplotype). L95 Lower 95% confidence interval, U95 Upper 95% confidence interval

Supplementary Table 4. Minor allele frequencies of SNPs with significant association with CBD from the discovery stage and older controls from Database for Genotypes and Phenotypes.

		Discovery stage		Replication stage		dbGaP datasets				PSP GWAS
**CHR**	**SNP**	**CBD (*n* = 152)**	**Controls (*n* = 3311)**	**CBD (*n* = 67)**	**Controls (*n* = 457)**	**APDGC (*n* = 991)**	**NGRC-PD (*n* = 1986)**	**NIA-LOAD (*n* = 743)**	**Overall (*n* = 3,720)**	**Stage 1 (*n* = 1115)**
2	rs963731	0.12	0.05	0.09	0.05	0.06	0.05	0.06	0.06	0.06
3	rs1768208	0.40	0.29	0.39	0.24	*na*	0.28[a]	0.27	0.31	0.36
7	rs1860743	0.20	0.11	0.09	0.12	*na*	*na*	0.13	0.28	0.12
8	rs643472	0.35	0.23	0.32	0.21	0.24	0.23	0.26	0.25	0.23
17	rs393152	0.08	0.24	0.07	0.25	0.20[b]	0.23[b]	0.24	0.22	0.06
17	rs242557	0.45	0.35	0.48	0.36	0.36	0.36	0.38	0.37	0.53
21	rs875125	0.17	0.10	0.05	0.08	0.11	0.09	0.08	0.09	0.10

Datasets were obtained from Database for Genotypes and Phenotypes (dbGaP) at http://www.ncbi.nlm.nih.gov/gap. Autopsy-Confirmed Parkinson Disease GWAS Consortium (991 controls) (APDGC), (phs000394.v1.p1); NeuroGenetics Research Consortium (1986 controls) (NGRC-PD), (phs000196.v2.p1); National Institute on Aging - Late Onset Alzheimer's Disease Family Study (743 controls) (NIA-LOAD), (phs000168.v1.p1). PSP GWAS Stage 1 allele frequencies are from those previously reported (reference 17 in the main text): Hoglinger, G.U. et al. Identification of common variants influencing risk of the tauopathy progressive supranuclear palsy. Nat. Genet. 43, 699-705 (2011). Minor allele frequencies (MAF) for top SNPs were not present in every dbGaP dataset; [a]MAF for rs545397 in LD with rs1768208 (R^2=0.94), [b]MAF for rs12185268 in LD with rs393152 (R^2=1), *na* not available.

Supplementary Table 5. Results from discovery stage GWAS in 152 CBD compared to 1986 control individuals from NeuroGenetics Research Consortium dbGaP dataset

				MAF			
**SNP**	**Chr**	**Gene**	**Discovery Stage Control Series**[a]	**Discovery Stage CBD Cases**	**Controls**	**OR (95% CI)**	***P***
rs12185268[b]	17q21	*MAPT*	CHOP	0.079	0.24	3.45 (2.29 - 5.34)	6.71×10^{-9}
			NGRC-PD		0.23	3.46 (2.27 - 5.28)	1.10×10^{-9}
rs643472	8p12	*lnc-KIF13B-1*	CHOP	0.35	0.23	1.88 (1.46 - 2.40)	7.12×10^{-7}
			NGRC-PD		0.23	1.83 (1.43 - 2.34)	1.24×10^{-6}
rs963731	2p22	*SOS1*	CHOP	0.12	0.054	2.46 (1.70 - 3.57)	2.04×10^{-6}
			NGRC-PD		0.051	2.42 (1.65 - 3.53)	2.70×10^{-6}
rs1768208	3p22	*MOBP*	CHOP	0.39	0.29	1.65 (1.30 - 2.09)	3.86×10^{-5}
			NGRC-PD		*na*	*na*	*na*
rs242557	17q21	*MAPT*	CHOP	0.45	0.35	1.48 (1.17 - 1.88)	1.50×10^{-3}
			NGRC-PD		0.36	1.45 (1.14 - 1.83)	2.05×10^{-3}
rs1860743	7q36	*PRKAG2*	CHOP	0.20	0.11	2.05 (1.52 - 2.78)	3.46×10^{-6}
			NGRC-PD		*na*	*na*	*na*
rs875125	21q22	*TSPEAR*	CHOP	0.17	0.10	2.02 (1.48 - 2.75)	7.92×10^{-6}
			NGRC-PD		0.093	2.06 (1.51 - 2.83)	4.46×10^{-6}

[a]Discovery stage (152 CBD, 1986 controls from dbGaP dataset NeuroGenetics Research Consortium (NGRC-PD), (phs000196.v2.p1) were analyzed by logistic regression under an additive model; genome inflation factor (λ=1.00). [b]The OR for rs12185268 is referencing the risk associated with the major allele (*MAPT* H1 haplotype). The protective allele (*MAPT* H2 haplotype) has an OR (95% CI) of 0.29 (0.19 – 0.44) in the Discovery Stage and 0.23 (0.11 – 0.46) in the Replication Stage. Genome-wide significant is defined as variants associated with $P < 1.6 \times 10^{-7}$ (315,542 markers) Chr, chromosome; MAF, minor allele frequency; OR, odds ratio; CI, confidence interval; lnc, long non-coding (RNA).

Supplementary Table 6. HaploReg v2 (http://www.broadinstitute.org/mammals/haploreg/) results for CBD susceptibility loci

rs643472 and variants with r2 ≥ 0.8

chr	pos (hg19)	LD (r^2)	LD (D')	variant	Ref	Alt	EUR freq	Enhancer histone marks	DNAse	Proteins bound	Motifs changed	GENCODE genes	dbSNP func annot
8	29153702	1	1	rs643061	C	T	0.74	HMEC, HepG2, NHEK, K562			SETDB1	18kb 3' of AC084262.2	
8	29153777	1	1	rs643472	C	T	0.74	HMEC, HepG2, NHEK, K562	HPDE6-E6E7,SAEC	STAT3	CCNT2,GATA,TAL1	17kb 3' of AC084262.2	
8	29153826	1	1	rs1708790	G	C	0.74	HMEC, HepG2, NHEK, K562	HMEC,NHEK,HPDE6-E6E7,RWPE1,pHTE, HEEpiC,PrEC,SAEC	STAT3	Hoxa7,Hsf	17kb 3' of AC084262.2	
8	29153879	0.99	1	rs1708791	G	C	0.74	HMEC, HepG2, NHEK, K562	HMEC,NHEK,HPDE6-E6E7,Osteobl,RWPE1 ,pHTE,HEEpiC,PrEC	STAT3	DMRT1,Irf	17kb 3' of AC084262.2	
8	29156462	0.93	-0.97	rs7834530	C	T	0.26				Pdx1	15kb 3' of AC084262.2	
8	29161883	0.91	-0.96	rs6998168	C	T	0.26				BCL,NF-I,Rad21,SMC3,Sin3Ak-20,THAP1,YY1,p300	9.4kb 3' of AC084262.2	
8	29162588	0.92	-0.96	rs6558107	T	C	0.26			POL2,NFYA	AP-1,Gfi1,Gfi1b,NF-Y,TATA	8.7kb 3' of AC084262.2	
8	29165243	0.88	-0.96	rs6996428	A	G	0.25				Bcl6b,Pax7,Sox	6kb 3' of AC084262.2	

rs1768208 and variants with r^2 >= 0.8

chr	pos (hg19)	LD (r^2)	LD (D')	variant	Ref	Alt	EUR freq	Enhancer histone marks	DNAse	Proteins bound	Motifs changed	GENCODE genes	dbSNP func annot
3	39508968	0.8	0.9	rs631312	G	A	0.69	H1	H9ES		CCNT2,Foxj1,Irf,Nanog,Sox,p300	*MOBP*	
3	39523003	1	1	rs1768208	T	C	0.69				PU.1,Tel2,p300	*MOBP*	intronic
3	39530083	0.96	1	rs545397	T	C	0.68		FibroP		SZF1-1,ZEB1	*MOBP*	intronic
3	39533759	0.95	0.99	rs1513219	T	C	0.68				NF-E2,SREBP	*MOBP*	intronic
3	39534481	0.99	0.99	rs616147	A	G	0.69				Ets	*MOBP*	intronic
3	39534742	0.95	0.99	rs1708104	T	C	0.68				DMRT2,Foxa,Foxc1,Foxd1,Foxj1, Foxk1,Foxl1,Foxo,TCF12	*MOBP*	intronic

rs963731 and variants with r^2 >= 0.8

chr	pos (hg19)	LD (r^2)	LD (D')	variant	Ref	Alt	EUR freq	Enhancer histone marks	DNAse	Proteins bound	Motifs changed	GENCODE genes	dbSNP func annot
2	39216873	1	1	rs963731	T	C	0.95				DMRT2,DMRT5,Dbx2,Dlx3,Gbx1, Gbx2,HNF1,Hoxa4,Hoxa9,Hoxd8, Msx-1,Ncx,Nobox,Pax-6,Pax7,Pou3f2	*SOS1*	intronic

**Supplementary Table 7. Neuropathologic diagnoses for samples used in eQTL studies**				
	**Cerebellum**		**Temporal Cortex**	
**Pathologic Diagnosis[a]**	***N* = 374**	**Relative %**	***N* = 399**	**Relative %**
Alzheimer's disease	197	53%	202	51%
Progressive supranuclear palsy (PSP)	98	26%	107	27%
Lewy Body Disease (LBD)	23	6%	25	6%
Corticobasal degeneration (CBD)	22	6%	22	6%
Frontotemporal lobar degeneration (FTLD)	15	4%	16	4%
Other	8	2%	10	3%
Multiple system atrophy (MSA)	7	2%	11	3%
Vascular dementia (VaD)	4	1%	6	2%

[a]All cases were evaluated by one neuropathologist (DWD).

**Supplementary Table 8. Discovery Stage power (%) to detect associations of SNPs and CBD status with 150 cases, 3000 controls for given odds ratios for different minor allele frequencies**												
**P-value**	*P*<0.05			*P*<0.01			*P*<0.001			$P<10^{-8}$		
**OR**	1.3	1.5	2	1.3	1.5	2	1.3	1.5	2	1.5	2	2.5
**Frequency of risk allele in controls**												
5%	21	47	87	10	28	74	3	11	52	<1	2	24
10%	33	64	98	14	42	93	4	22	83	<1	12	65
20%	49	85	99	28	66	<99	10	40	98	1	40	94
30%	55	90	<99	32	76	<99	13	51	99	1	53	98
50%	57	91	<99	34	79	<99	13	54	99	1	40	94
70%	50	85	<99	24	65	99	6	31	92	<1	4	37
80%	35	72	99	14	44	92	3	18	69	<1	<1	1
90%	20	41	85	5	16	57	<1	2	16	<1	<1	<1
95%	12	18	48	1	3	14	<1	<1	<1	<1	<1	<1

This power analysis assumes that genotypes are distributed according to Hardy-Weinberg equilibrium in 150 cases and 3000 controls separately and that analysis is conducted with logistic regression. The odds ratio (OR) refers to an increase of one copy of the minor allele. Power assessed with 1000 simulations of each scenario.

Supplementary Table 9. Replication Stage power (%) to detect associations of SNPs and CBD status with 60 cases, 700 controls for given odds ratios for different minor allele frequencies

***P*-value**	*P*<0.05				*P*<0.001			
**OR**	1.3	1.5	2	2.5	1.3	1.5	2	2.5
**Frequency of risk allele in controls**								
5%	12	21	55	79	3	8	31	61
10%	14	33	75	95	5	14	55	85
15%	21	41	85	98	8	21	68	94
20%	23	49	90	99	8	26	74	97
25%	26	51	95	100	8	28	82	98
30%	28	54	95	99	12	28	83	98
75%	20	40	80	94	5	15	50	78

This power analysis assumes that genotypes are distributed according to Hardy-Weinberg equilibrium in 60 cases and 700 controls separately and that analysis is conducted with logistic regression. The odds ratio (OR) refers to an increase of one copy of the minor allele. Power assessed with 1000 simulations of each scenario.
